# Characterization of antibodies in single-chain format against the E7 oncoprotein of the Human papillomavirus type 16 and their improvement by mutagenesis

**DOI:** 10.1186/1471-2407-7-25

**Published:** 2007-01-31

**Authors:** Maria Gabriella Donà, Colomba Giorgi, Luisa Accardi

**Affiliations:** 1 Section of Molecular Pathogenesis, Department of Infectious, Parasitic and Immunomediated Diseases, Istituto Superiore di Sanità, Rome, Italy

## Abstract

**Background:**

Human papillomaviruses (HPV) are the etiological agents of cervical cancer. The viral E7 protein plays a crucial role in viral oncogenesis. Many strategies have been explored to block the E7 oncoprotein activity. The single-chain variable antibody fragments (scFvs) are valuable tools in cancer immunotherapy and can be used as "intracellular antibodies" to knock out specific protein functions. For both in vivo and in vitro employment, the scFv intrinsic solubility and stability are important to achieve long-lasting effects. Here we report the characterization in terms of reactivity, solubility and thermal stability of three anti-HPV16 E7 scFvs. We have also analysed the scFv43 sequence with the aim of improving stability and then activity of the antibody, previously shown to have antiproliferative activity when expressed in HPV16-positive cells.

**Methods:**

The three anti-HPV16 E7 scFv 32, 43 51 were selected from the ETH-2 "phage-display" library. Thermal stability was evaluated with ELISA by determining the residual activity of each purified scFv against the recombinant HPV16 E7, after incubation in the presence of human seroalbumine for different time-intervals at different temperatures. Sequence analysis of the scFvs was performed with BLAST and CLUSTALL programs. The scFv43 aminoacid changes were reverted back to the consensus sequence from the immunoglobuline database by site-directed mutagenesis. ScFv solubility was evaluated with Western blotting by determining their relative amounts in the soluble and insoluble fractions of both prokaryotic and eukaryotic systems.

**Results:**

ScFv51 was the most thermally stable scFv considered. Sequence analysis of the most reactive scFv43 has evidenced 2 amino acid changes possibly involved in molecule stability, in the VH and VL CDR3 regions respectively. By mutagenesis, two novel scFv43-derived scFvs were obtained, scFv43 M1 and M2. ScFv43 M2 showed to have improved thermal stability and solubility in comparison with the parental scFv43.

**Conclusion:**

The characterization of 5 specific anti-HPV16 E7 scFvs shows features important for their activity *in vivo*. ScFv43 M2 shows higher thermal stability with respect to the parental scFv43, and scFv51 shows high stability and solubility. These properties make the 2 scFvs the best candidates to be tested for anti-E7 activity *in vivo*.

## Background

Human papillomaviruses (HPVs) cause one of the most common sexually transmitted infections in the world. A subset of "high-risk" HPV genotypes is unequivocally associated to cervical cancer, the second main cause of death for cancer in women worldwide [1,2]. Nowadays the impending commercialisation of the prophylactic anti-HPV vaccine is shifting research efforts towards tumour therapy. Many efforts have been made to develop effective treatments for the HPV-associated lesions [3]. The delivery of antitumoral agents to the cervical cancer cells may represent a valid strategy for their treatment especially at an early lesion stage, and in addition or alternative to surgery of the advanced lesions.

The viral proteins E6 and E7, which play a crucial role in viral oncogenesis [4-6] are recognised "tumor-specific antigens" and are therefore considered suitable targets for either immunotherapy or therapeutic vaccination against the HPV-associated tumors [7].

Recombinant single-chain variable fragments (scFv) antibodies represent powerful tools for different immunotherapy purposes and are particularly suitable in intracellular immunisation to knock out specific protein functions [8-11]. Many of them are either in clinical trial or successfully used in therapy.

The good potential of scFvs for biomedical use is severely limited by their intrinsic solubility and stability, which are important characteristics to achieve long-lasting effects both *in vitro *and *in vivo *[12]. However, it is worth noting that the *in vitro *and *in vivo *scFv specificities are not always comparable to one another. ScFv solubility and stability are related to their primary structure and mostly depend on the intrinsic ability of correctly folding by forming intra-chain disulphide bonds in reducing environments, in both prokaryotic and eukaryotic cells [13,14]. The scFv thermal stability is a decisive property for their applications to targeted tumor therapy [15]. In fact the scFvs must be stable at 37°C for many hours to be able to penetrate into tumors, an activity that can take 12 hours or more [16].

We have previously reported the selection of three different scFvs (scFv 32, 43 and 51) against the E7 oncoprotein of the HPV16 (16E7) from the ETH-2 phage display library of human antibody fragments [17]. We have described the antiproliferative effect of the most reactive scFv43, when it is expressed in the nuclear and secretory compartments of the HPV16-positive cervical carcinoma SiHa cell line [18]. We have also demonstrated that this effect could be specifically ascribed to inhibition of the E7 activity, in promoting cell proliferation.

In this paper we have investigated the biophysical properties of the three anti-E7 scFvs when these are expressed in either prokaryotic or eukaryotic systems, in the attempt to focus parameters important for their activity both *in vitro *and *in vivo *[19].

To establish if the differences observed in the scFv stability could be related to their secondary structure, we have compared the scFv amino acid sequences to the consensus sequences of both the IMGT database and the validated intrabody database (VIDA) of intracellular stable scFvs [20,21]. Two main non-synonymous and possibly destabilizing mutations were identified in the scFv43 sequence. A mutagenesis strategy was designed to partially revert these mutations back to the original sequence, obtaining 2 novel antibodies, scFv43 M1 and scFv43 M2.

The 5 anti-16E7 scFvs, 2 obtained by mutagenesis and 3 originally selected, were compared to one another in terms of thermal stability and solubility in both prokaryotic and eukaryotic systems.

## Methods

### ScFvs selection

The anti-HPV16E7 scFvs were selected from the ETH-2 phage display library of human antibody fragments after three rounds of panning in solution against the recombinant His-HPV16E7 protein as already described by Accardi et al. [18].

### Cloning of scFv32 and 51 sequences in scFvExpress vectors and plasmids

The scFv32 and 51 coding sequence were isolated from pDN332 by NcoI/NotI restriction and subcloned into the vectors scFvE-cyto and scFvE-nuclear, digested with the same enzymes. For cloning into scFvE-sekdel, the encoding sequences were PCR-amplified (95°C for 1 minute, 50°C for 1 minute, 74°C for 1 minute, 35 cycles) using the SalsekD-NotsekR2 couple of primers. The sequences of the primers used are the following (restriction sites are underlined):

SalsekD 5'CGGCGTCGACCCGAGGTGCAGCTGGTGG 3'

NotsekR2 5'CGGCGCGGCCGCTTTGATTTCCACCTTGGTCCC 3'

PCR products were double-digested with SalI/NotI, gel-purified using GFX PCR DNA and Gel Band Purification Kit (Amersham Biosciences) and ligated into scFvE-sekdel digested with the same enzymes. The ligation products were used to transform competent XL1-blue *E. coli *cells (Stratagene). The scFv fragments isolated with the described procedure and cloned into the scFvExpress vectors do not retain the FLAG-tag and the His-tag sequences present in the original plasmid, and acquire a c-myc-tag at their C-terminus, utilised for detection. The R4-cyto construct expressing an anti-β-gal scFv targeted to the cytoplasm, utilised as a control of stable and soluble scFv antibody, was kindly provided by A. Cattaneo.

### Sequence alignments

Sequence alignments to NCBI and to VIDA were carried out using Immunoglobulin BLAST and CLUSTALL programs.

### Protein expression and purification from periplasmic and total extracts

The His-E7 expression and purification from the JM109 *E. coli *strain and the scFv expression from the HB2151 *E. coli *strain have been already described by Accardi et al. [18]. Briefly, 1 ml-aliquots of each bacterial culture were collected just before isopropylthio-β-galactoside (IPTG) induction (non induced samples), and at 2–4 hours post-induction, pelletted and resuspended in 100 μl of 2× SDS-loading buffer. For each sample, 20 μl-aliquots of both the pellet and the supernatant were analysed for the scFv expression by Western blotting followed by chemiluminescence as described below. For scFv expression in the M15 *E. coli *strain, an overnight (ON) culture was diluted 1: 40 in 2xTY 2%, glucose/amp (100 μg/ml)/Kan (25 μg/ml), grown until OD_600_= 0.8, and centrifuged to harvest bacteria. The pellet was recovered in 1l of 2xTY/amp/2 mM IPTG and the culture grown for 4 hours at room temperature (RT). The bacteria recovered from this culture after centrifugations were treated differently to prepare periplasmic extract (PE) and total extract (TE). For PE preparation, the bacteria were resuspended in 50 ml TES (Tris-HCl pH = 7, 20% saccarose, 1 mM EDTA) in the presence of protease inhibitors (Roche). After centrifugation at 4000 rpm for 40' at 4°C, the supernatant (PE1) was kept at 4°C and the pellet resuspended in 50 ml of MgSO_4_, shaken 10' at RT, and centrifuged at 10000 rpm for 20' at 4°C; the supernatant (PE2) was harvested after addition of protease inhibitors and combined to PE1, obtaining PE. TE extracts were prepared by resuspending the bacteria recovered from 1 litre-culture in 50 ml of lysis buffer (50 mM Tris-HCl pH = 7, 1% Triton X-100, 1 mM EDTA, 1 mM EGTA, 0.5 M NaCl) containing Complete EDTA-free protease inhibitors (Roche), sonicated and incubated for 20' on ice. After centrifugation at 12,000 g for 10' at 4°C, supernatant (TE) was recovered and used for the scFv purification. The scFv purification was performed by affinity chromatography either on Ni-NTA beads (Qiagen) or on protein A-Sepharose CL-4B (Amersham Biosciences), according to the manufacturers' instructions. Purity of the proteins was evaluated by Coomassie Blue Staining after SDS-PAGE.

### ScFv reactivity and affinity determination

The scFv reactivity and affinity for 16E7 were evaluated by Western blot analysis and ELISA as already described by Accardi et al. [18]. Briefly, the competitive ELISA for affinity determination was performed by incubating in solution the purified scFvs at concentration of 0.7 μg/ml, with His-E7 (competitor) at different concentrations in the range 10^-10 ^and 10^-6 ^M, for 2 hours. The samples were then incubated ON in microtiter wells coated with 300 ng/well His-E7, at 4°C; the reaction was revealed by the anti-FLAG M2 mAb (Sigma St. Louis, MO) followed by GAM-HRP IgG (Amresco, Solon, OH) and using TMB substrate kit for peroxidase (Vector Laboratories, Inc. Burlingame, CA) colorimetric evaluation. The affinity constant was defined as the reciprocal of the competitor concentration required to reduce of the 50% the binding of the antibody not preincubated with E7, and is expressed in M^-1^.

### ScFv thermal stability

Purified scFvs were diluted in Dulbecco's PBS (DPBS) + Ca^2+ ^+ Mg^2+ ^containing 0.2% human serum albumin (HSA), and incubated either at 37°C for predefined intervals of time or at 40°-50°-60°C for 10'. After incubation, the antibodies were assayed for their ability of binding the E7 protein by ELISA. Coating of a microtiter 96-wells plate (Nunc Polysorp) was performed ON at 4°C with purified recombinant His-E7 at 300 ng/well in carbonate/bicarbonate buffer (Pierce, Rockford, IL). The plate was saturated with 2% NFDM and then incubated with the purified scFvs at the desired dilutions ON at 4°C. Immunocomplexes were detected using the anti-FLAG M2 mAb (Sigma, St. Louis, MO) at the dilution 1:3000, followed by GAM-HRP IgG (Amresco, Solon, OH) as above described.

### ScFv43 mutagenesis

Mutations in scFv43 were obtained by PCR amplification of the scFv43 sequence using QuickChange™ Site-Directed Mutagenesis Kit (Stratagene) and the following primers (the mutated nucleotides are underlined):

43M1 5' ctgtcagcagcgtcatggtaatccggc 3'

43M1 5' gccggattaccatgacgctgctgacag 5'

43M2 5' gcagctatgccacgagctgggtccgcc 3'

43M2 5' ggcggacccagctcgtggcatagctgc 3'

The PCR reaction was performed as follows: 95°C for 30 seconds, 45°C for 1 minute; 68°C for 9 minutes, 18 cycles. The PCR products were used to transform competent DH5α cells (Stratagene). Plasmids DNA from the obtained clones as extracted using Qiagen Spin Kit (Qiagen) and sequenced to confirm the incorporation of the desired mutations. ScFv43 M1 and scFv43 M2 plasmids were used to transform competent HB2151 cells (Stratagene) for scFv production.

### Cell lines and transfection

The cervical epithelial tumour SiHa cell line (ATCC HTB-35), harbouring the HPV16 genome, and the SV40-transformed African green monkey kidney Cos-7 cells (ATCC CRL 1651), were used in this study. Both the SiHa and Cos-7 cells were maintained in Dulbecco's modified Eagle's medium (DMEM) containing 10% heat-inactivated fetal calf serum, 100 units/ml penicillin, 100 μg/ml streptomycin and 2 mM glutamine. Transient transfection of 80% confluent cells was performed using the recombinant scFvExpress plasmids and Lipofectamine 2000 (Invitrogen) according to the manufacturer's recommendations. ScFv expression in SiHa cells was analysed 48 hours post-transfection by Western blotting as described below.

### Analysis of the scFv expression

The identity of the scFvs expressed in prokaryotic systems was evaluated after separation on SDS-PAGE and blotting onto PVDF membrane, by Western blot analysis using the anti-FLAG M2 monoclonal antibody (mAb; Sigma, St. Louis, MO) as a primary antibody followed by a goat anti-mouse horseradish peroxidase-conjugated (GAM-HRP)) IgG (Amresco, Solon, OH).

Analysis of scFv expression in eukaryotic systems was performed by Western blotting after separation by SDS-PAGE of cell lysates in 2 × SDS loading buffer and blotting onto PVDF membrane. The rabbit anti-c-myc mAb, clone 9E10 (Sigma) was used as a primary antibody followed by a GAR-HRP-conjugated IgG (Cappel, Amresco, Solon, OH) incubation. The immunocomplexes were revealed by Chemiluminescence using the Super Signal West Pico Chemiluminescent Substrate (Pierce, Rockford, IL).

### Preparation and analysis of the soluble and insoluble extracts

Soluble and insoluble cellular extracts were obtained as described in ref. [11]. Briefly, a 100 mm tissue culture dish of Cos-7 cells, transfected with each of the scFvE-cyto recombinant vectors, was lysed with 300 μl ice-cold extraction buffer containing containing Tris-Cl 20 mM, pH 8, MgCl2 20 mM, 0.5% NP40, 0.1 mg/ml leupeptin, chymostatin, and aprotinin, and 0.1 mM PMSF for 15 minutes. Cellular extracts were centrifuged at 15,000 rpm for 15 minutes at 4°C to separate soluble (supernatant) from insoluble proteins (pellet). Twenty μl of protein A-Sepharose in Tris-Cl 25 mM, pH 8.6, NaCl 150 mM (TBS) were incubated ON at 4°C with 2 μg of rabbit anti-c-myc mAb clone 9E10, washed three times with TBS, pH 8.0, and centrifuged for 1 minute at 1500 rpm. Three hundred μl of the soluble pool were incubated with 20 μl of 9E10-protein A-Sepharose for 2 hours at 4°C. The Sepharose beads were washed four times with TBS containing 0.1% NP40 and once with Tris-Cl 5 mM, pH 7.6. After addition of sample buffer (Tris-Cl 125 mM, pH 6.8, SDS 1%, glycerol 5%, DTT 10 mM, and bromophenol blue 0.005%), boiling for 2 minutes, and centrifugation for 1 minute (15,000 rpm, RT), the supernatants were loaded onto a 10% SDS-polyacrylamide gel and analysed by Western blotting as above described.

## Results

### ScFvs expression, production and solubility in prokaryotic cells

**Figure 1:**
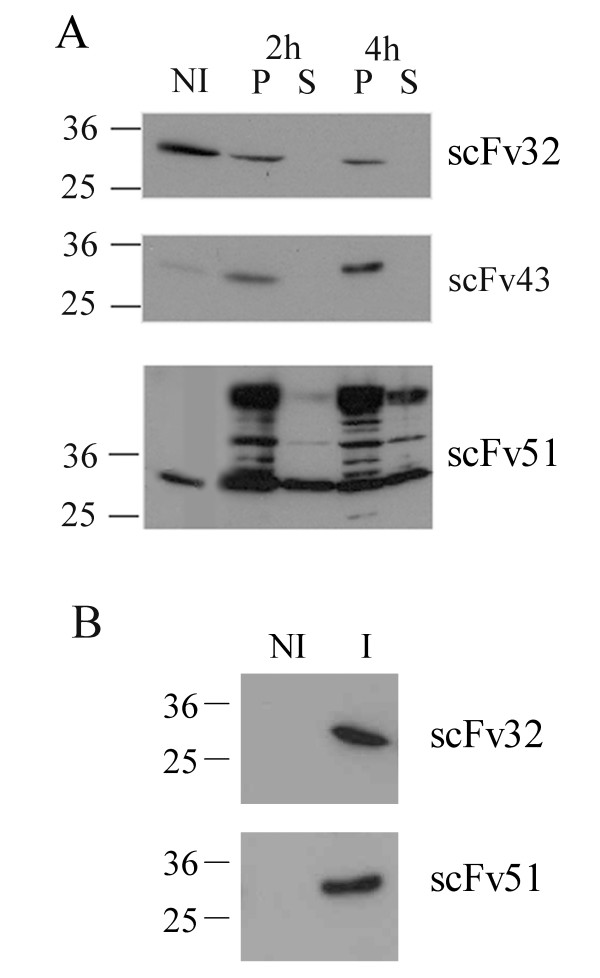
Expression of scFv 32, 43 and 51 in *E. coli*. Panel A. Western blot analysis of lysates from cultures of the HB2151 *E. coli *strain expressing the scFv 32, 43 and 51, non induced (NI) or IPTG-induced for 2 and 4 hours at RT, before lysis. For the non induced cultures the total bacterial lysate was analysed, whereas pellet (P) and supernatant (S) fractions from the IPTG-induced cultures were utilized. Panel B. Western blot analysis of the total lysates from the M15/Rep+ *E. coli *strain expressing the scFv 32 and 51, non induced (NI) and IPTG-induced (I) for 4 hours at RT. The M15/Rep+ *E. coli *strain was used to repress the antibody expression in the non induced cultures. The Western blotting was performed using the anti-FLAG M2 mAb as a primary antibody, followed by GAM-HRP incubation. The immune complexes were revealed by chemiluminescence. Molecular mass markers are shown on the left.

The specific anti-16E7 scFvs 32, 43 and 51, representative of three different groups of scFvs recently selected from the ETH-2 library [18] were expressed in the HB2151 *E. coli *strain. In inducible prokaryotic systems, the yield of expressed product can be influenced by its gene repression efficiency before induction. The amount of scFv 32, 43 and 51 products present in both the pellet and the culture medium before and after IPTG induction was evaluated by Western blotting. As shown in Fig. 1A, the scFv43 expression was quite well repressed in the non induced culture (NI), in contrast with that of scFv32 and scFv51. The last one was the most expressed and its production was improved by IPTG induction. At both 2 and 4 hours of induction all the scFvs were found in the bacterial pellet obtained by centrifugation of the cell culture (P) and just scFv51 was found also in the culture medium (S). As expected, the three scFvs were released into the culture medium after ON induction (not shown).

The amount of scFv product obtained was usually very low, especially for scFv43, therefore different strategies were employed to maximize the scFv expression before purification. With the aim of efficiently repressing the scFv 32 and 51 expression in the non induced cultures, the scFvs were expressed in the M15/Rep+ *E. coli *strain. This strain carries the pREP 4 repressor plasmid inducing a strong repression of the lacZ-promoter. A complete repression was obtained for both the scFvs (Fig. 1B). The expression from different colonies of each scFv clone was analysed after IPTG induction and the best expressing colonies were selected and used for antibody production (not shown). Parameters like temperature and composition of the culture medium were also considered to optimise the production. IPTG induction at RT with the addition of sorbitol to the culture medium (Materials and Methods and ref [22]) was found to be the optimal condition for the expression of soluble scFvs.

**Figure 2:**
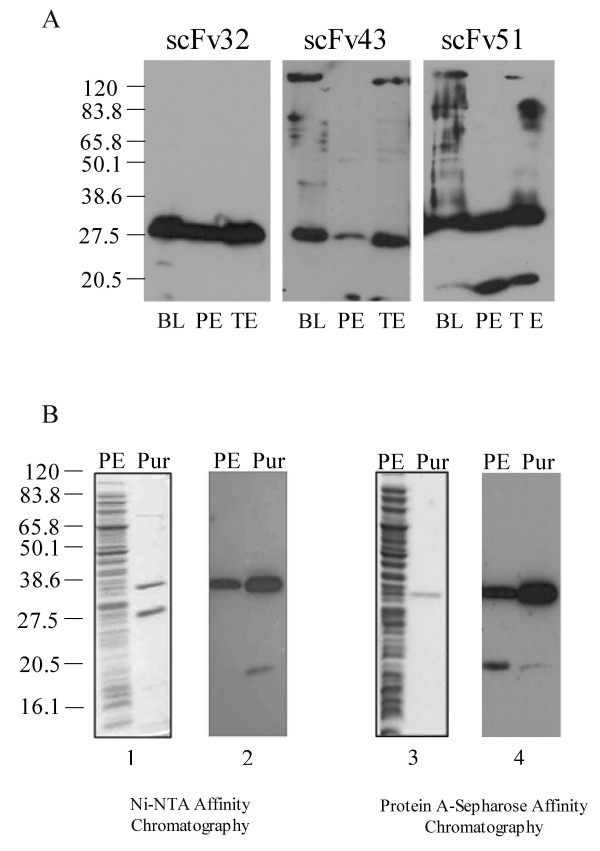
Panel A. Western blot analysis of scFv 32, 43 and 51 bacterial lysates (BL), periplasmic extracts (PE) and soluble total extracts (TE) obtained from equal volumes of bacterial culture after IPTG induction for 4 hours at RT. Panel B. Comassie-blue stained gel after SDS-PAGE (1 and 3) and Western blot analysis (2 and 4) of the periplasmic extract (PE) and of the purified product (Pur) from cultures of M15 *E. coli *strain expressing the scFv32 as an example. Purification was performed either by NiNTA or Protein A-Sepharose affinity-chromatography as indicated. The scFv detection was performed by the anti-FLAG tag M2 mAb followed by GAM-HRP incubation. Molecular mass markers are shown on the left.

In prokaryotic systems, the scFvs that are expressed in a soluble form are secreted into the periplasm thanks to the PelB leader sequence fused to their N-terminus, whereas the scFvs that are intrinsically insoluble remain inside the cytoplasm. To investigate eventual differences of solubility among the scFvs, we compared the scFv amount present in bacterial lysates (BL), which include both soluble and insoluble proteins, in the periplasmic extract (PE), and in the total extract of soluble proteins obtained after mild lysis by sonication (TE). The Western blot analysis clearly showed that the major part of scFv43 remained inside the cytoplasm, while just a small amount was secreted in the periplasm (Fig. 2A). No significant difference between the reactivities of the scFv43 extracted from TE and PE could be revealed either in ELISA or in Western blotting (data not shown). In contrast, scFv 32 and 51 were equally distributed between the PE and TE fractions, showing a higher solubility of these antibody fragments with respect to scFv43 (Fig. 2A). The Western blot analysis demonstrated that a huge quantity of scFv51 was secreted in the culture medium. The possibility to purify the antibody fragment from this fraction was then explored, but the reactivity of the scFv obtained was 0.5-0.1 times less of that purified from the periplasm (data not shown). These results indicate that the highest amount of functional antibodies was obtainable starting from TE fraction for scFv43 [18] and from PE fraction for scFv 32 and 51.

The scFvs, carrying a His-tail fused to the C-terminus, were purified by Ni-NTA affinity chromatography and analysed by SDS-PAGE followed by Western blotting performed using a mAb against the FLAG-tag positioned upstream the His-tail. The Comassie-Blue Stained Gel analysis of all the three purified scFvs showed a unique band with the expected molecular weight of 27 KDa together with an unexpected band of lower molecular mass not detectable in Western blotting by the anti-FLAG mAb. In Fig. 2B the results obtained using the purified scFv32 are shown. The nature of this protein was uncertain: it may either derive from cleavage of the FLAG-tag positioned downstream the antibody sequence or be a contaminant His-containing bacterial protein. To test the second hypothesis, the scFv purification was performed by affinity chromatography on protein A-Sepharose, a resin which can bind the DP47-derived VH regions, bypassing the absence of the Fc region in the antibody. The purified products exhibited high purity indicating that the smaller protein was a bacterial contamination (Fig. 2B, panels 3 and 4). Owing to its specificity and reliability, this procedure was then utilized for the purification of all the three scFvs.

### ScFvs thermal stability

To evaluate the scFv thermal stability, a crucial parameter for their *in vivo *activity; aliquots of purified scFvs were incubated for different intervals of time at 37°C in the presence of HSA, simulating the *in vivo *environment [23,24]. After each interval of time, the scFv residual reactivity against the recombinant 16E7 protein was measured by ELISA. The time at which the OD_450 _value was 50% (t_1/2_) of that at time 0, was used as a parameter of thermal stability.

**Figure 3:**
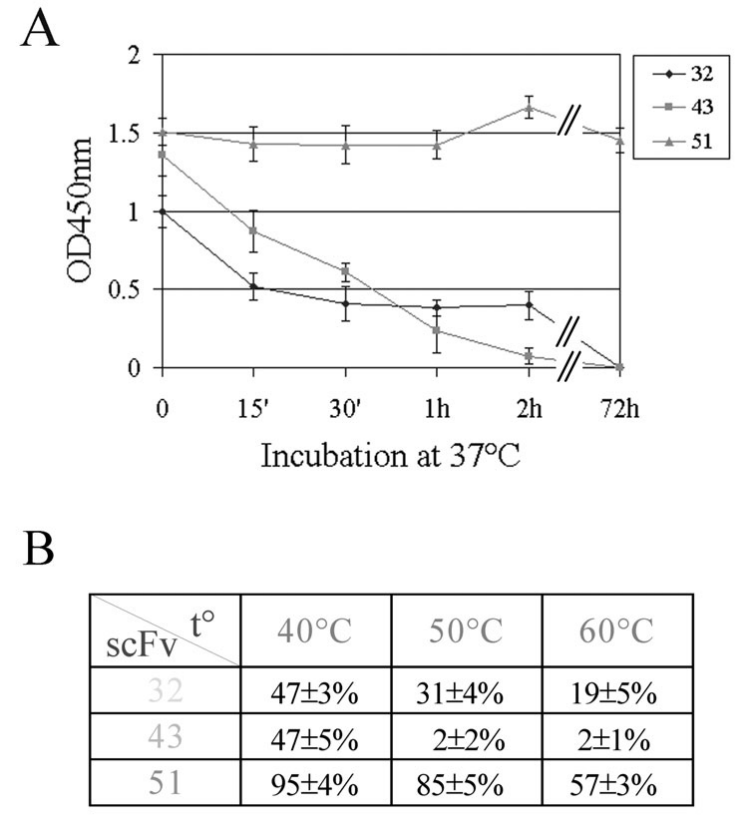
Analysis of the thermal stability of the scFv 32, 43 and 51. The residual anti-E7 reactivity of the scFvs was evaluated by ELISA after incubation at 37°C for the indicated intervals of time (A), and (B) at 40°-50°-60°C for 10'.

In Fig. 3A the OD_450 _values after incubation at 37°C for 15 minutes, 30 minutes, 1 hour, 2 hours and 72 hours, are reported for each of the scFvs; it can be observed that the t_1/2 _is 15 minutes for scFv32 and about 30 minutes for scFv43, while for scFv51 the t_1/2 _could not be determined because it was outside the time interval examined. Different trends of scFv reactivity can be observed. The scFv32 reactivity halved between time 0 and 15 minutes, and then remained stable at least up to 6 hours incubation (data not shown); in contrast, the scFv43 reactivity was near 0 after just 2 hours, whereas scFv51 exhibited a quite constant OD_450 _value up to 72 hours.

The scFv thermal stability was also analysed by checking the residual scFv reactivity after incubation at temperatures >37°C for 10 minutes (Fig. 3B). Interestingly, the scFv43 reactivity was reduced to 47% of the initial value after incubation at 40°C and was almost abolished (2%) after incubation at 50°C; the scFv32 reactivity halved at 40°C and was greatly reduced at 50°C, whereas the scFv51 reactivity remained almost the same at 50°C and halved just at 60°C. These results indicate scFv51 to be the most thermally stable among the three antibody fragments.

### Sequence analysis

The phage display library of scFvs used in this study was constructed from the DP47, DPL16 and DPK 22 germ line genes. In a previous study we showed that the VH regions of the three scFvs derived from the DP47 germ line gene whereas the scFv 32 and 51 VL derived from the DPL16, and the scFv43 VL from the DPK22 [18].

**Figure 4:**
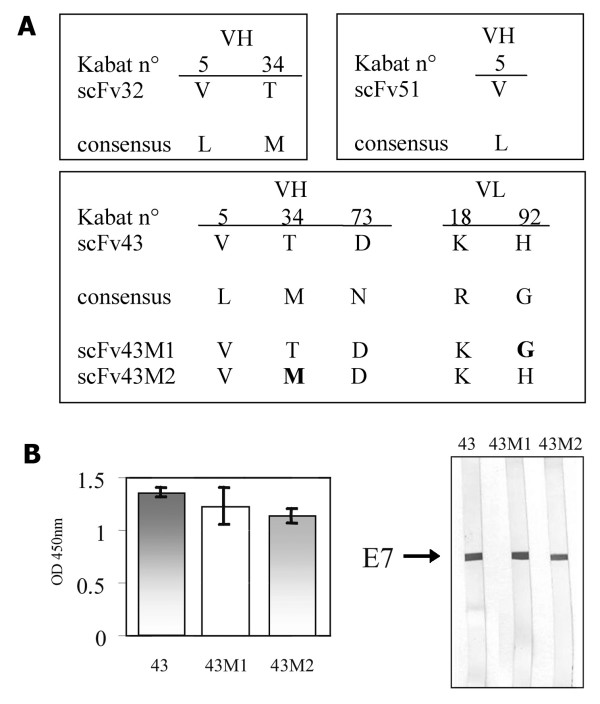
Panel A. ScFv sequence alignment and mutagenesis design. The scFv 32, 43 and 51 aminoacidic sequences were aligned to the consensus sequences of the respective germ-line genes, namely DP47 for the VH region of the three antibodies, DPL16 for the VL of scFv32 and 51 and DPK22 for the VL of scFv43. The positions of the mismatching amino acids are indicated for the three scFvs; the numbering is according to Kabat and Wu [25]. The amino acids mutated in the scFv43M1 and M2 sequences with respect to that of scFv43 are in bold. Panel B. Comparison of the scFv43, 43M1 and 43M2 reactivity against E7 protein. The purified scFvs have been utilized at a concentration of 0.7 μg/ml in ELISA (left hand) and of 1 μg/ml in Western blotting (right hand). The E7 is indicated by an arrow. The immunocomplexes were revealed by anti-FLAG tag mAb followed by GAM-HRP incubation.

To investigate the presence of significant amino acid substitutions in the selected scFvs, we have compared their nucleotidic and deduced aminoacidic sequences to the immunoglobulin heavy chain (IGHV) consensus sequences from the ImMunoGeneTics (IMGT) database. The mismatchings found in the aminoacid sequences are shown in Fig. 4 (first line of the three panels). A sense mutation resulting in the substitution of Leucine by Valine at position VH5 (numbering according to ref. [25]) is present in the nucleotide sequences of all the three scFvs; a sense mutation causing the replacement of Methionine with Threonine at position VH34 is present in both scFv 32 and scFv43. Another sense mutation, resulting in the change from Asparagine to Aspartate at position VH73, is present just in the scFv43 sequence. The last mutation is found in 38% of the Ig VH sequences analysed by Chothia and collaborators [26] and seems not to be relevant for the molecule stability.

Looking at the scFv43 VL, an aminoacid diversity (from Glycine to Histidine) has been found in the CDR3 at position VL92. Of interest, this position was left unchanged during the ETH-2 library construction as it is considered relevant for the scFv stability [17]. Consequently, it can be hypothesized that a mutation has arisen during the amplification cycles following the scFv selection.

The residues VH34 and VH73 belong to the 76 residues that form the "common core" of the IgG variable domains [26]. The residue at position 34 is part of the "common core" buried structure, important for the conformation of the loops in the hypervariable region. Furthermore, the VH34 residue is included in the CDR2, which forms the antigen-binding region together with the CDR1 and the CDR3. The VH34 amino acid is hydrophobic in 95% of the IgGs analysed by Chothia and collaborators. Notably, at this position, scFvs 32 and 43 have a Threonine, a polar residue present in just 0.1% of the sequences of the IMGT database; therefore this amino acid could play a role in inducing a conformational change of the scFv molecules.

### Construction and characterization of the scFv43 mutants

ScFv43 has been previously shown to inhibit cell proliferation when expressed in nuclear and secretory compartments but not in cell cytoplasm of the HPV16-positive SiHa cells. We hypothesized that the lack of effect could be ascribed either to the low amount of E7 present in the cytoplasm or to the low stability of the antibody fragment in this reducing environment, where it tends to form "aggresomes" [18]. The results above reported actually demonstrate that scFv43 is rather instable *in vitro*. On the other hand, this antibody fragment is an interesting molecule because of its antiproliferative activity. In the attempt to improve its performance by increasing stability, the scFv43 sequence was mutated to partially revert it back to that of the germ-line gene consensus sequences. Utilizing a site-directed mutagenesis approach, two scFv43 revertants were constructed: scFv43 M2 and M1. In the scFv43 M2 sequences, the Threonine at position VH34 was replaced by a Methionine; in the scFv43 M1 sequences, the Histidine at position VL92 was replaced by a Glycine (Fig. 4). Notably, the aminoacids restored at positions VH34 and VL92 of the IgG consensus sequences, are the same present at the corresponding positions in the consensus sequences for intracellular stable antibodies of the VIDA data bank [20].

The biophysical properties of the mutants have been then characterized and compared to those of scFv43. The reactivity against the 16E7 antigen was perfectly comparable to that of the parental scFv43 (not shown). A different affinity could be expected particularly for scFv43 M1, which was mutated in the CDR3 region. However, the affinity of the two revertants was 10^6 ^M^-1^, in the same range of the parental scFv43.

**Figure 5:**
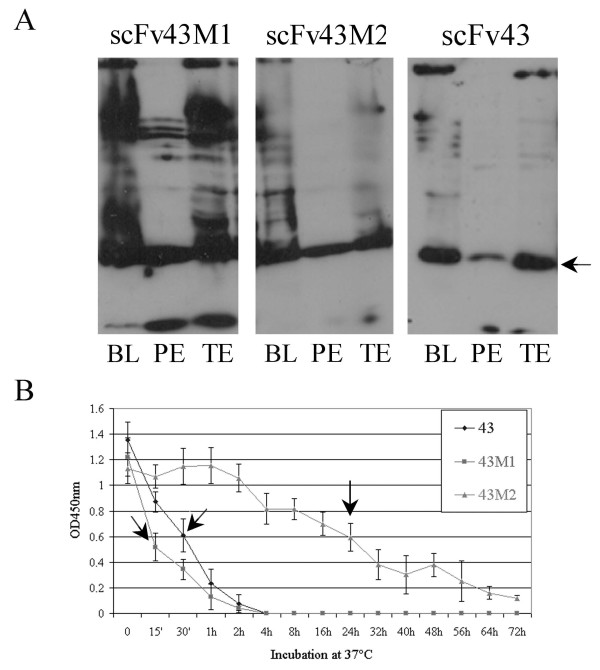
Panel A. Western blot analysis of the bacterial lysates (BL), periplasmic extracts (PE) and soluble total extracts (TE) obtained from equal volumes of bacterial cultures expressing the scFv 43 M1 and M2 mutants, and scFv43. The Western blotting was performed using the anti-FLAG M2 mAb as a primary antibody, followed by GAM-HRP IgG incubation. The immunocomplexes were revealed by chemiluminescence. The molecular mass markers are indicated on the left. The scFv molecular weight of about 27.5 KDa is indicated by an arrow. Panel B. Analysis of the scFv 43 M1 and scFv43 M2 thermal stability in comparison with that of the parental scFv43. The scFvs were incubated at 37°C for different time intervals, indicated on the abscissa. Note that the scale is not linear. At the end of the incubation the residual reactivity towards the recombinant E7 protein was evaluated by ELISA; the OD values at 450 nm are reported on the ordinate. The t_1/2, _defined as the time of halving of the anti-E7 reactivity after incubation at 37°C, is indicated by an arrow for each scFv.

Solubility in bacteria and thermal stability of the revertants were then analysed as above described for scFv 32, 43 and 51, and compared to those of scFv43. As shown in Fig 5A, both scFv43 M1 and M2 were equally distributed between the PE and TE fractions, showing solubility in the periplasm superior to that of scFv43. In addition, Fig. 5B shows the t_1/2 _of scFv43 M2 to be 24 hours, much higher than that of scFv43 (30 minutes). In contrast, the scFv43 M1 t_1/2 _was 15 minutes, even lower than that of the parental scFv43. All together these results suggest that the aminoacid at position VH34 is involved in the scFv stability.

### ScFvs intracellular expression and stability in cellular compartments

To examine the expression of scFv 32, 51 and 43M2 in eukaryotic systems, the sequences encoding the three scFvs were cloned into the vectors of the scFvExpress (scFvE) series [27,28], which direct the expressed proteins to different cell compartments. The vector scFvEcyto for cytoplasmic localization does not contain any targeting signal, the scFvEnuc contains a nuclear localization signal, and the scFvESekdel contains both a secretory leader and a Sekdel sequence for retention in the endoplasmic reticulum. Three recombinant plasmids were obtained for each scFv that were used to transfect the HPV16-positive SiHa cells.

**Figure 6:**
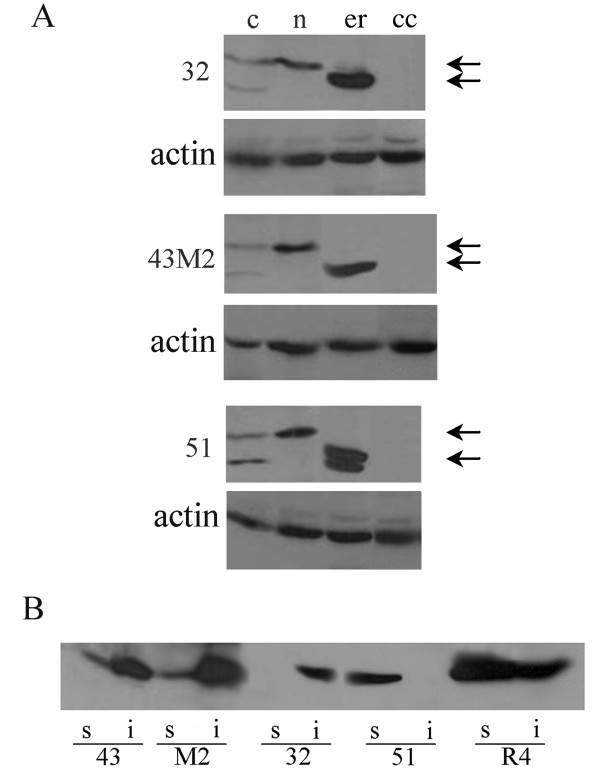
Analysis of the scFv expression in eukaryotic cells. Panel A. Western blot analysis of extracts from the HPV16-positive SiHa cells transfected with plasmids expressing the scFv 32, 43M2 and 51, targeted to the cellular cytoplasm (lane c), nucleus (lane n) and endoplasmic reticulum (lane er). The analysis was performed 48 hours after transfection. Non transfected cell lysates were used as negative control. The Western blotting was performed using the rabbit anti-c-myc mAb (clone 9E10) followed by GAM-HRP IgG incubation and chemiluminescence. The scFvs are indicated by arrows. To control for protein loading, the blots were probed with a mouse anti-actin mAb (Sigma). Panel B. Analysis of the scFv solubility. Cos-7 cells were transfected with plasmids expressing in the cell cytoplasm the scFv 43 (43), 43M2 (M2), 32, 51 and the highly stable and soluble R4 anti-β-gal scFv as a control. The cellular extracts were fractionated in soluble (lanes s) and insoluble (lanes i) fractions. The presence of the antibodies in these fractions was examined by Western blotting performed as above described.

Expression of the scFvs in these cells was monitored by Western blotting (Fig. 6A), and their correct intracellular localization was confirmed by immunofluorescence (data not shown). All the three scFvs were expressed at the highest level in the endoplasmic reticulum, and at the lowest level in the cytoplasm. The scFv32, 51 and 43M2 seem to have the same expression pattern as that previously observed for scFv43. In fact this scFv was expressed at a high level in the secretory compartment and to a lesser extent in the cytoplasm [18]. To examine if the scFv cytoplasmic low level could be related to their low solubility in this eukaryotic environment, Cos-7 cells were transfected with the different scFv constructs including the new scFv43 M2 targeting the cytoplasm, and the scFv solubility was analysed by cell fractioning [11]. The Cos-7 cells are HPV-negative cells and were chosen because they can be transfected at a higher efficiency than SiHa cells.

The scFv amount present in the soluble versus insoluble cell fractions was analysed by Western blotting. An anti-β-gal scFv recognized to be highly stable and soluble, R4, was used as a control [29]. The results shown in Fig 6B indicate that scFv43 and scFv43 M2 have the same distribution between the soluble and insoluble fractions with the larger antibody amount present in the insoluble fraction. This result suggests that the revertant antibody seems not to have acquired a better solubility compared to the parental one. The scFv32 and 51 exhibit a totally different pattern of solubility; the first scFv seems to be completely insoluble, while the other one is mainly present in the soluble fraction.

## Discussion

In this paper we have analysed and compared the biophysical properties of three anti-16 E7 scFvs (scFv 32, 43 and 51) in view of their possible use *in vivo *for therapy of the HPV-associated lesions. These scFvs exhibited different yield and solubility when expressed in prokaryotic system. ScFv43 resulted to be the least soluble, with the major part of expressed product remaining inside the bacterial cell cytoplasm, and just a small amount secreted into the periplasm. To achieve a sufficient production, different strategies were adopted. The scFv43 was extracted from the total cell lysate whereas the other two antibodies were extracted from the periplasm. The purified scFvs produced by the different procedures exhibited a good reactivity against the recombinant antigen [18].

The thermal stability is a very important characteristic of the scFvs in view of their possible use *in vivo*. ScFv 43 and 32 have shown the same behaviour during the first hour of incubation at 37°C but the scFv43 was completely loosing its reactivity during the second hour of incubation, while the scFv32 reactivity, after the initial drop, remained stable up to 6 h. The scFv51 was the most stable antibody fragment because maintained its initial reactivity at least up to 72 hours of incubation.

In spite of its low thermal stability, the scFv43 seemed to work efficiently in hampering the E7 activity in the HPV16-positive SiHa cells, as we have reported in a previous study [18]. These apparently conflicting results prompted us to investigate the reasons of this instability. The scFv stability depends on sequence contributions of both the framework and the CDRs, which are responsible for the antigen binding. Also, a mutation in one single residue of the framework can play an important role in maintaining the structure of the whole molecule [30]. Many strategies have been adopted to improve stability of intrabodies for extracellular and intracellular applications [31-33].

The comparison of the deduced scFv amino acid sequences with both the amino acid sequences of the Igs from the IMGT Database and the stable intracellular scFv consensus sequences of the VIDA database [20] revealed some mismatching. At position VH5 of all the three scFv sequences, a sense mutation is present (Leu to Val, both neutral amino acids, numbering according to ref. [25]).

At position VH34, scFv32 and scFv43 have a sense mutation which causes a change from a neutral to a polar amino acid (Met to Thr). ScFv43 presents additional mismatching at residues VH73 (Asp to Asn, both hydrophilic amino acids) and VL92 (Gly to His, from a neutral to a basic amino acid).

ScFv51 is the antibody fragment that shows the highest thermal stability and this result is in agreement with the identity of its framework sequences to the IgG sequences from the IMGT Database.

It is interesting to note that the VH34 residue is positioned in the Ig common core [26]. The substitution of a hydrophobic with a hydrophilic amino acid in this region, as is the case of scFv32 and scFv43, may cause modifications in the molecule conformation. However, when attempting a correlation between the differences observed in the thermal stability of these two scFvs and their amino acid sequences, one has to consider that the VL regions of the two scFvs are derived from different germ-line genes.

Of interest, the scFv43 amino acids at positions VH 34 and 73 are not "fitting" the consensus sequence of the VIDA database, based on the scaffold of intrabodies with improved solubility and expression properties *in vivo *[20,21]. A mutagenesis strategy has been designed to possibly improve the scFv43 stability and solubility by reverting the amino acids back to those present in the consensus sequence of both the IgG and the VIDA databases. The final aim was to improve the scFv43 intracellular performance without affecting its antigen-binding activity.

The amino acid at position VL92 resulted not to be important for the scFv43 stability. In contrast, reverting position VH34 seems to have fulfilled the goal, with scFv43 M2 showing improved features of thermal stability with respect to scFv43, as assessed by the shifting of t_1/2 _from 30 minutes to 24 hours.

## Conclusion

In summary, the specific anti-16E7 scFvs described in this paper exhibit characteristics which are important for their functionality in eukaryotic systems. The thermal stability of scFv43, an antibody fragment able to contrast the E7 activity, has been improved, and we could expect scF43 M2 to function better than the parental scFv43. The scFv51 shows a high stability, a property often more important than affinity for the antigen in determining intracellular behaviour and efficacy, together with a high solubility. These properties render the two scFvs best candidates to be tested for anti-E7 activity *in vivo*.

## Abbreviations

scFv, single chain variable fragment; HPV, human papillomavirus; Ig, immunoglobulin; VL, light chain variable region; VH, heavy chain variable region; CDR, complementarity determining region; mAb, monoclonal antibody; HSA, human seroalbumine; anti-β-gal, anti-β-galactosidase; IPTG, isopropylthio-β-D-galactoside; GAM-HRP, goat anti-mouse horseradish peroxidase-conjugated; OD, optical density; ON, overnight; RT, room temperature; PVDF, polyvinilidene difluoride; GAR-HRP, goat anti-rabbit horseradish peroxidase-conjugated; GAR-FITC, goat anti-rabbit fluorescein-labeled; VIDA, validated intrabody database.

## Competing interests

The author(s) declare that they have no competing interests.

## Authors' contributions

GMD participated in the study design, acquisition and interpretation of data and revision of the manuscript. CG participated in the interpretation of data and revision of manuscript. LA participated in the study design, acquisition and interpretation of data, coordinated the study, and drafted up the manuscript.

All authors read and approved the final manuscript.

## Pre-publication history

The pre-publication history for this paper can be accessed here:



## References

[B1] Bosch FX, de Sanjosé S (2003). Chapter 1: human papillomavirus and cervical cancer-burden and assessment of causality. J Natl Cancer Inst Monogr.

[B2] Munoz N, Bosch FX, de Sanjose S, Herrero R, Castellsague X, Shah KV, Snijders PJ, Meijer CJ, International Agency for Research on Cancer Multicenter Cervical Cancer Study Group (2003). Epidemiologic classification of human papillomavirus types associated with cervical cancer. N Engl J Med.

[B3] Eiben GL, da Silva DM, Fausch SC, Le Poole IC, Nishimura MI, Kast WM (2003). Cervical cancer vaccines: recent advances in HPV research. Viral Immunol.

[B4] Munger K, Basile JR, Duensing S, Eichten A, Gonzalez SL, Grace M, Zacny VL (2001). Biological activities and molecular targets of the human papillomavirus E7 oncoprotein. Oncogene.

[B5] zur Hausen H (2002). Papillomaviruses and cancer: from basic studies to clinical application. Nat Rev Cancer.

[B6] Balsitis SJ, Sage J, Duensing S, Munger K, Jacks T, Lambert PF (2003). Recapitulation of the effects of the human papillomavirus type 16 E7 oncogene on mouse epithelium by somatic Rb deletion and detection of pRb-independent effects of E7 in vivo. Mol Cell Biol.

[B7] Cornelison TL (2000). Human papillomavirus genotype 16 vaccines for cervical cancer prophylaxis and treatment. Curr Opin Oncol.

[B8] Cardinale A, Lener M, Messina S, Cattaneo A, Biocca S (1998). The mode of action of Y13-259 scFv fragment intracellularly expressed in mammalian cells. FEBS Lett.

[B9] Lener M, Horn IR, Cardinale A, Messina S, Nielsen UB, Rybak SM, Hoogenboom HR, Cattaneo A, Biocca S (2000). Diverting a protein from its cellular location by intracellular antibodies. The case of p21Ras. Eur J Biochem.

[B10] Holliger P, Hudson PJ (2005). Engineered antibody fragments and the rise of single domains. Nature Biotechnology.

[B11] Cardinale A, Filesi I, Vetrugno V, Pocchiari M, Sy MS, Biocca S (2005). Trapping prion protein in the endoplasmic reticulum impairs PrPC maturation and prevents PrPSc accumulation. J Biol Chem.

[B12] Worn A, Pluckthun A (2001). Stability engineering of antibody Single-chain Fv fragments. J Mol Biol.

[B13] Sibler AP, Nordhammer A, Masson M, Martineau P, Trave G, Weiss E (2003). Nucleocytoplasmic shuttling of antigen in mammalian cells conferred by a soluble versus insoluble single-chain antibody fragment equipped with import/export signals. Exp Cell Res.

[B14] Sibler AP, Courtete J, Muller CD, Zeder-Lutz G, Weiss E (2005). Extended half-life upon binding of destabilized intrabodies allows specific detection of antigen in mammalian cells. FEBS J.

[B15] Willuda J, Honegger A, Waibel R, Schubiger PA, Stahel R, Zangemeister-Wittke U, Pluckthun A (1999). High thermal stability is essential for tumor targeting of antibody fragments: engineering of a humanized anti-epithelial glycoprotein-2 (epithelial cell adhesion molecule) single-chain Fv fragment. Cancer Res.

[B16] Jain RK (1988). Determinants of tumor blood flow: a review. Cancer Res.

[B17] Pini A, Viti F, Santucci A, Carnemolla B, Zardi L, Neri P, Neri D (1998). Design and use of a phage display library. Human antibodies with subnanomolar affinity against a marker of angiogenesis eluted from a two-dimensional gel. J Biol Chem.

[B18] Accardi L, Dona MG, Di Bonito P, Giorgi C (2005). Intracellular anti-E7 human antibodies in single-chain format inhibit proliferation of HPV16-positive cervical carcinoma cells. Int J Cancer.

[B19] Zhu Q, Zeng C, Huhalov A, Yao J, Turi TG, Danley D, Hynes T, Cong Y, DiMattia D, Kennedy S, Daumy G, Schaeffer E, Marasco WA, Huston JS (1999). Extended half-life and elevated steady-state level of a single-chain Fv intrabody are critical for specific intracellular retargeting of its antigen, caspase-7. J Immunol Methods.

[B20] Visintin M, Settanni G, Maritan A, Graziosi S, Marks JD, Cattaneo A (2002). The intracellular antibody capture technology (IACT): towards a consensus sequence for intracellular antibodies. J Mol Biol.

[B21] Visintin M, Quondam M, Cattaneo A (2004). The intracellular antibody capture technology: towards the high-throughput selection of functional intracellular antibodies for target validation. Methods.

[B22] Sandee D, Tungpradabkul S, Kurokawa Y, Fukui K, Takagi M (2005). Combination of Dsb coexpression and an addition of sorbitol markedly enhanced soluble expression of single-chain Fv in Escherichia coli. Biotechnol Bioeng.

[B23] Lu D, Jimenez X, Witte L, Zhu Z (2004). The effect of variable domain orientation and arrangement on the antigen-binding activity of a recombinant human bispecific diabody. Biochem Biophys Res Commun.

[B24] Hugo N, Weidenhaupt M, Beukes M, Xu B, Janson JC, Vernet T, Altschuh D (2003). VL position 34 is a key determinant for the engineering of stable antibodies with fast dissociation rates. Protein Eng.

[B25] Kabat EA, Wu TT (1991). Identical V region amino acid sequences and segments of sequences in antibodies of different specificities. Relative contributions of VH and VL genes, minigenes, and complementarity-determining regions to binding of antibody-combining sites. J Immunol.

[B26] Chothia C, Gelfand I, Kister A (1998). Structural determinants in the sequences of Immunoglobulin Variable Domain. J Mol Biol.

[B27] Persic L, Righi M, Roberts A, Hoogenboom HR, Cattaneo A, Bradbury A (1997). Targeting vectors for intracellular immunisation. Gene.

[B28] Persic L, Roberts A, Wilton J, Cattaneo A, Bradbury A, Hoogenboom HR (1997). An integrated vector system for the eukaryotic expression of antibodies or their fragments after selection from phage display libraries. Gene.

[B29] Martineau P, Betton JM (1999). In vitro folding and thermodynamic stability of an antibody fragment selected in vivo for high expression levels in Escherichia coli cytoplasm. J Mol Biol.

[B30] Tramontano A, Chothia C, Lesk AM (1990). Framework residue 71 is a major determinant of the position and conformation of the second hypervariable region in the VH domains of immunoglobulins. J Mol Biol.

[B31] Ewert S, Honegger A, Pluckthun A (2004). Stability improvement of antibodies for extracellular and intracellular applications: CDR grafting to stable frameworks and structure-based framework engineering. Methods.

[B32] Ewert S, Honegger A, Pluckthun A (2003). Structure-based improvement of the biophysical properties of immunoglobulin VH domains with a generalizable approach. Biochemistry.

[B33] Ewert S, Huber T, Honegger A, Pluckthun A (2003). Biophysical properties of human antibody variable domains. J Mol Biol.

